# Spin reporting is common in pilot and feasibility trials in hip and knee arthroplasty: a methodological analysis of a cross-sectional sample

**DOI:** 10.1186/s40814-026-01846-2

**Published:** 2026-05-30

**Authors:** Zinnia Chung, Leigh-Ann Grant, Jeslyn Chen, Lawrence Mbuagbaw, Lipalo Mokete, Lehana Thabane

**Affiliations:** 1 Michael G. DeGroote School of Medicine, McMaster University, Hamilton, ON Canada; 2 Department of Health Research Methods, Evidence and Impact, McMaster University, Hamilton, ON Canada; 3 Department of Anesthesia, McMaster University, Hamilton, ON Canada; 4 Department of Pediatrics, McMaster University, Hamilton, ON Canada; 5 Biostatistics Unit, Father Sean O’Sullivan Research Centre, St. Joseph’s Healthcare, Hamilton, ON Canada; 6 Centre for Development of Best Practices in Health (CDBPH), Yaoundé Central Hospital, Yaoundé, Cameroon; 7 Division of Epidemiology and Biostatistics, Department of Global Health, Stellenbosch University, Cape Town, South Africa; 8 Division of Orthopaedics, University of the Witwatersrand, Johannesburg, South Africa; 9 Research Institute of St Joeseph’s Hamilton, St Joseph’s Healthcare Hamilton, Hamilton, ON Canada; 10 Faculty of Health Sciences, University of Johannesburg, Johannesburg, South Africa

**Keywords:** Pilot and feasibility trials, Pilot trials, Feasibility studies, Hip arthroplasty, Knee arthroplasty, Hip replacement, Knee replacement, Methodological analysis

## Abstract

**Background:**

Pilot and feasibility trials help identify methodological and logistical challenges. However, biased reporting, known as “spin,” may distort overall study findings and mislead readers, presenting content through a subjective lens. Accurate assessments of feasibility are critical in orthopedic research, where the continued popularity of procedures such as hip and knee arthroplasties emphasizes a need for rapid advancement through research and development. The prevalence of spin practices among pilot and feasibility trials in hip and knee arthroplasties remains unclear.

To evaluate the prevalence of spin reporting practices in pilot and feasibility trials focused on hip and knee arthroplasty. The secondary objective is to identify factors associated with the level of spin featured in the analyzed manuscripts.

**Methods:**

A search of PubMed identified 147 trials published between 2017 and 2023, selected using stratified random sampling. Studies were screened for the presence of three spin criteria defined in previous literature, and summarized descriptively.

**Results:**

Spin appeared in 88.4% of studies (95% CI 83.3–93.6). Emphasis on intervention effectiveness (81.6%) and statistical significance rather than feasibility (60.5%) were the most common types. Spin was frequent in abstracts (78.9%) and discussions (79.6%), with the highest rates also observed in privately funded (95.7%) and pharmacologic (100%) studies. No clear trend was observed over time.

**Conclusions:**

Spin is widespread in pilot and feasibility trials involving hip and knee arthroplasty, particularly in abstracts and discussions. Greater adherence to reporting guidelines and explicit communication of feasibility objectives and study limitations are necessary to improve transparency and reduce interpretive bias in this field.

**Graphical Abstract:**

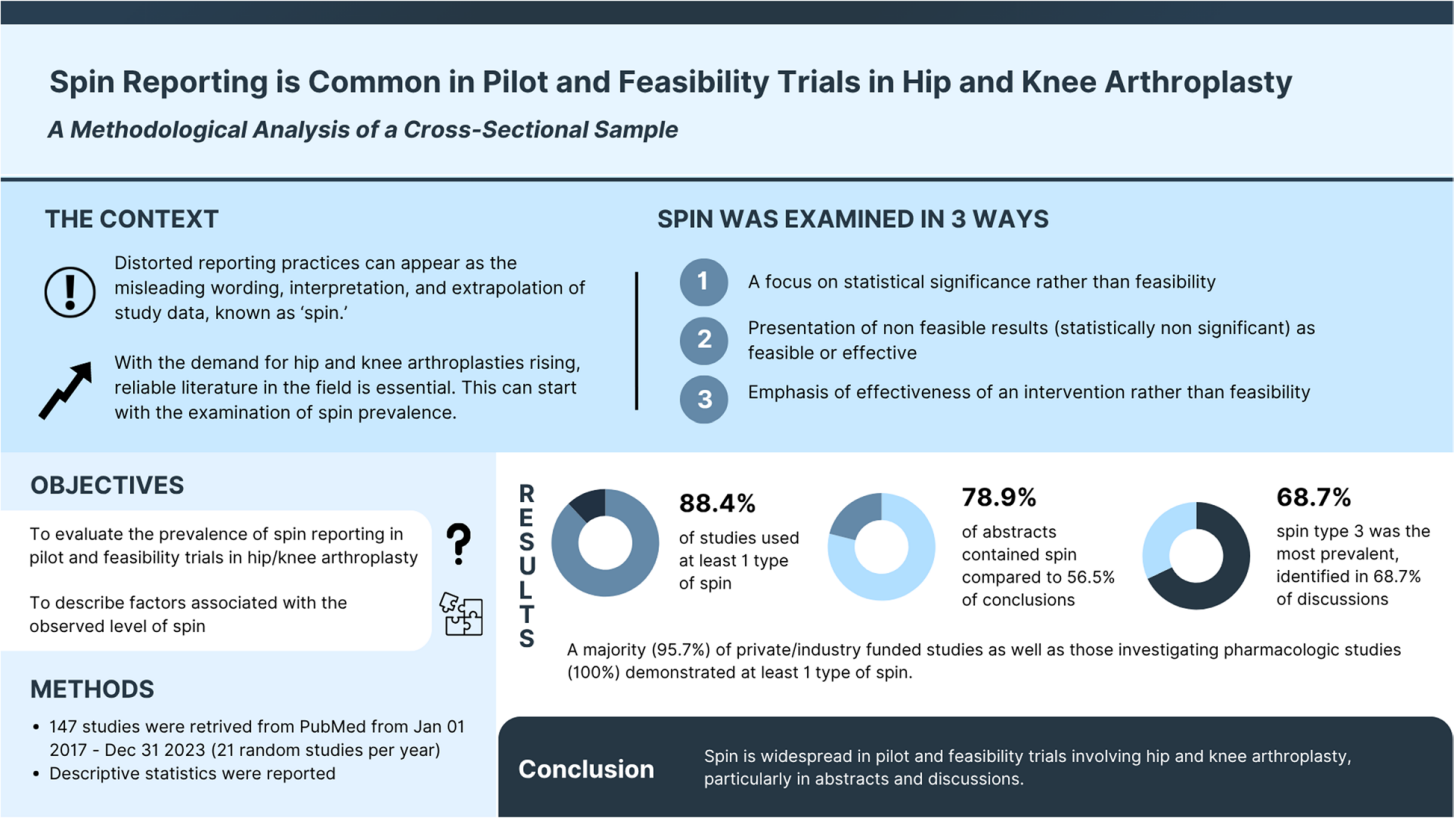

## Key messages

### Issues being addressed

- Pilot and feasibility studies are fundamental to research advancement; unfortunately, there is a scarcity of relevant guidelines for the standardization of reporting.
- As such, suboptimal or incomplete reporting is common, coupled with inconsistent literature quality and unclear reporting completeness in hip and knee arthroplasty pilot and feasibility trials.

### What we know about the topic

- “Spin” is defined as misleading reporting practices, including focusing on statistical significance rather than feasibility, presenting non-feasible results as feasible/effective, and emphasizing efficacy/benefits over feasibility.
- The prevalence of spin in the reporting of pilot and feasibility trials has not been sufficiently explored in orthopedic literature, but has been notable in biomedical research.

### Key findings from this study

- Consistent with other biomedical studies, misleading reporting practices were prevalent. At least one type of spin was detected in 88.4% of the included manuscripts, especially in the form of emphasizing potential efficacy or intervention benefits rather than feasibility.
- Specific report sections had greater prevalence of spin than others: i.e. abstracts (78.9%) and discussions (79.6% of studies), had a greater spin prevalence than conclusions (56.5%).

### Implications

- This study outlines the importance of improved communication of feasibility objectives and study limitations in future pilot and feasibility studies, through adherence to reporting guidelines such as the CONSORT extension for pilot trials.

## Background

Pilot and feasibility studies are crucial for optimizing, assessing, and scaling research studies for success. By evaluating project designs on a smaller scale, these trials help researchers identify key barriers to execution and assess core elements such as recruitment potential, study retention, and the assessment process [1]. In particular, randomized controlled trials (RCTs) that included pilot trials were found to achieve publication more quickly, and in higher-impact journals [2]. Additional benefits, such as a lower risk of bias in concealment and masking procedures, have also been identified, demonstrating the direct role of pilot trials in enhancing study design [2]. However, these benefits are contingent on pilot trial outcomes being reported in an objective manner, which should highlight both design strengths and limitations. This ensures that pilot and feasibility trials can maximize their functionality, providing an accurate assessment of a study’s design while identifying areas where uncertainty may still exist [3].

Unfortunately, distorted reporting practices can compromise result interpretation, significantly diminishing the utility of trial outcomes [4]. These approaches often manifest as misleading reporting, interpretation, and extrapolation of study data, referred to as “spin” tactics [4]. Boutron et al. further defined several forms of spin, including approaches that focus on statistical significance, interpret non-significant data as comparable in effectiveness, or make claims of benefit despite statistically non-significant results [5]. Other examples of spin are made evident through the use of overly strong wording choices. For instance, in the field of oncology, 56% of studies from a sample of 62 publications used leading terms when reporting results (title, abstract, results, etc.) [6]. Other spin tactics include reporting results as being “significant”, “optimal”, or “novel” inappropriately. Meanwhile, in a study of 128 non-randomized intervention papers, 84% demonstrated at least 1 example of spin within their abstracts, while a review of cardiovascular literature found spin in 67% of published main texts [7, 8]. This appears to be a common pattern across disciplines, with a high prevalence of spin also identified in an analysis of traumatic brain injury, even among projects published in leading medical journals [9].

Inaccurate reporting practices often manifest as an overemphasis on certain study findings while minimizing others. This misrepresentation of pilot/feasibility trial results can subconsciously influence how readers interpret the findings. Although further research is needed to assess the specific impact of spin on readers’ decision-making, two commonly noted consequences are the misinterpretation and favorable presentation of unfeasible results [4]. In this context, misleading communication in study reports calls into question the reliability of pilot and feasibility studies. Furthermore, while the prevalence of spin practices has been studied in published biomedical literature (encompassing clinical trials, observational studies, meta-analyses, systematic reviews, and diagnostic accuracy studies), its impact in pilot trials has yet to be fully explored [4]. In orthopedics, for example, where the demand for hip and knee arthroplasties is projected to increase by almost 40% in 2060, ongoing research supported by pilot and feasibility trials will be crucial [10].

Therefore, the primary objective of this study is to evaluate the prevalence of spin reporting practices in pilot and feasibility trials focused on hip and knee arthroplasty, based on the types of spin identified by Boutron et al. and McKechnie et al. [5, 11]. Specifically, spin can be categorized into (1) a focus on statistical significance rather than feasibility, (2) the presentation of non-feasible results (statistically non-significant) as feasible or effective, and (3) the emphasis of effectiveness or potential intervention benefits rather than feasibility [5, 11]. The secondary objective is to describe factors associated with the level of spin featured in the analyzed manuscripts.

## Methods

### Study design

This is a methodological analysis of a cross-sectional sample of articles. Of note, the population used in this study was previously analyzed in a separate publication focused on completeness of reporting based on adherence to the CONSORT 2010 extension for pilot and feasibility trials (10.1136/bmjopen-2024-085441). Neither spin nor any factors associated with its prevalence have been investigated or reported using this dataset. As this study is based on published, publicly available data without the involvement of human participants, ethics review board approval was not required.

### Eligibility criteria

Eligible studies were published between January 01 2017 and December 31, 2023, covering 7 years. This timeframe was chosen based on the 2016 publication of the Consolidated Standards of Reporting Trials (CONSORT) extension for randomized pilot and feasibility trials, ensuring a more standardized population for analysis [12]. Manuscripts were included if they were published/available in the English language and explicitly described themselves as “pilot” or “feasibility” studies in their title, abstract, or main text. This review focused on primary research, with eligible designs encompassing randomized and non-randomized clinical trials, surveys, mixed methods, and observational cohort, cross-sectional, and case–control approaches. Secondary research, including case reports, review articles, and meta-analyses were excluded. Additionally, eligible studies were focused on hip and knee arthroplasty, with specific emphasis on perioperative clinical outcomes (preoperative, intraoperative, or postoperative). No fixed postoperative timeframe was imposed, provided they were directly attributable to the index surgical procedure. Relevant topics included but were not limited to surgical approaches, imaging, rehabilitation, or biomedical equipment. Only studies involving living human participants were considered; those using animal populations, artificial models, or isolated specimens were not considered.

### Search strategy

A manual search of the PubMed database from January 01, 2017, to December 31, 2023, was conducted. Relevant texts were identified using a systematic search strategy, using keywords and Medical Subject Headings such as “feasibility studies,” “pilot projects,” “arthroplasty, replacement knee” and “arthroplasty, replacement, hip”. Studies classified as either pilot or feasibility were included in the sample. The full search strategy may be found in Appendix 1, under PubMed Search Strategy.

### Study selection

Microsoft Excel was used to host the database search results after duplicate entries were filtered out. All texts were screened by three reviewers (ZC, LG, JC) in accordance with the inclusion and exclusion criteria. This process involved three separate phases, with the screening process first limited to titles, then abstracts, and finally to the full-text manuscripts. Any discrepancies among the three reviewers were resolved by consensus. A fourth reviewer (LT) was also available to address any further disagreements requiring additional consultation. Following the initial screening process, a stratified random sample was obtained from each of the examined years (2017–2023) to ensure appropriate representation across the specified timeframe. A sample size of 21 studies per year allowed for an equal distribution of studies in the context of volume limitations within the selected database. Concurrently, the randomization process was completed using a digital random number generator.

### Data extraction and synthesis

Data collection and extraction were conducted using a form on Microsoft Excel. This form was developed by all three reviewers and included basic study characteristics such as the date of publication, the continent of the first author, the presence of a structured abstract, and the sample size used (based on the intention to treat). Specific factors found to be associated with the prevalence of spin, such as study design, intervention type, published journal, and source of funding were also recorded [4, 13, 14].

The prevalence of spin was based on the presence of any spin types identified by Boutron et al. and McKechnie et al. [5, 11]. The three types included (1) a focus on statistical significance rather than feasibility, (2) the presentation of non-feasible results (statistically non-significant) as feasible or effective, and (3) the emphasis of effectiveness or potential intervention benefits rather than feasibility [5]. Furthermore, the frequency at which each spin type appeared was reported alongside its location in the text (abstract, main text, or conclusion). Spin criteria that did not appear were indicated with a “0” and those that were not applicable were marked with “n/a.” Based on the level of spin reported, studies could be classified under “high spin” (2 or more spin tactics, and a prevalence of spin above the 75th percentile), “moderate spin” (spin prevalence around the 50th percentile) or “low spin” (spin prevalence below the 25th percentile to no spin at all). All data were extracted by three independent reviewers (ZC, LG, JC). Each study was assigned to two reviewers and extracted in duplicate to ensure independent verification. Any discrepancies were reviewed and resolved by a fourth reviewer (LT). The JBI risk of bias assessment tool was used to evaluate the studies similarly by two independent reviewers (ZC, LG), who also worked in duplicate [15]. Discrepancies were addressed by a third reviewer (JC).

### Statistical analysis

Spin outcomes were recorded using descriptive statistics, captured as the number of studies (%) reporting of each type of spin, alongside the prevalence of spin across the study population. The percentage of studies found to have at least one of the item definitions of ‘spin,’ in their abstracts, results/discussions, and conclusions was also reported in this way. Data was also organized visually to assess patterns associated with the prevalence of spin, sorting by source of funding (federal, private, not-for-profit, none, and not specified), the intervention type (physiotherapy, surgical, diagnostic, pharmacologic, equipment, or other), the year of publication (2017–2023), and the size of the study population (divided into quartiles, with groupings of 2–21, 22–35, 36–61, 62+) [4, 13, 14]. Analyses were performed using Microsoft Excel computing software and results were tabulated and presented using Microsoft Word.

## Results

### Search results

**Fig. 1:**
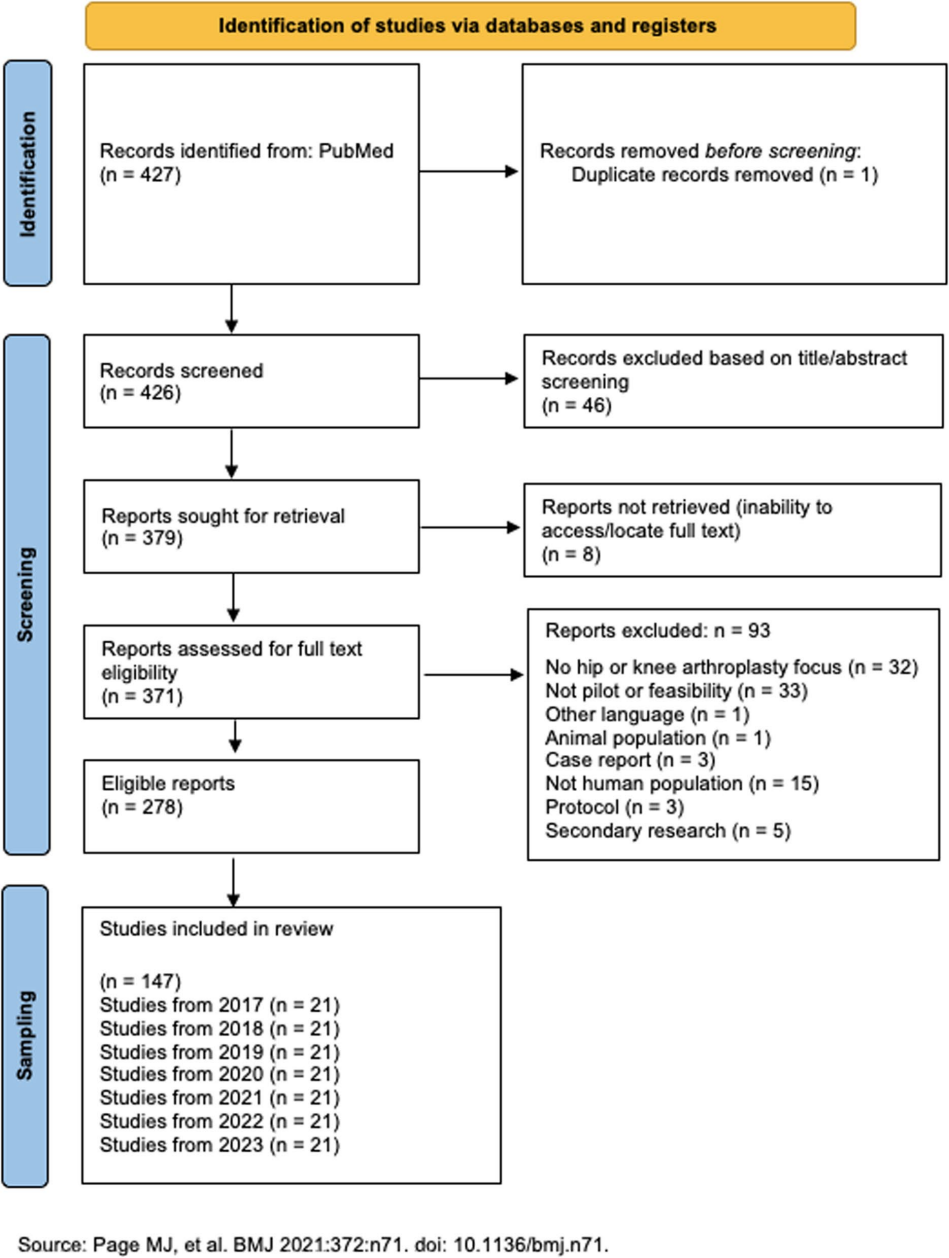
Literature screening and selection flow diagram

Four hundred twenty-seven records were returned from the PubMed database, 371 of which were retrieved for further assessment following preliminary screening of titles and abstracts. After full-text screening, 278 eligible publications were identified, with a stratified random sample of 21 studies selected from each included year (2017–2023). The literature screening and selection flow diagram is featured in Fig. 1.

### Study characteristics

The characteristics of the study population (*n* = 147) are summarized in Table 1, including study design, primary focus, intervention type and category, sample size, and funding source. randomized controlled trials accounted for the largest proportion (35.4% (52/147), followed by cohort studies (25.9%, 38/147). A majority of studies focused on knee arthroplasty (53.1%, 78/147) while 27.2% (40/147) examined hip procedures. Physiotherapy-based interventions were the most frequently investigated (19.7%, 29/147), followed by surgical (15%, 22/147) as well as diagnostic (15%, 22/147) interventions. Most interventions targeted the post-operative phase (52.4, 77/142), and enrolled smaller samples, with 34% (50/147) including between 2 and 25 participants. In terms of funding, 35.4% (52/147) of studies received no financial support, while others were funded by federal (26.5%, 39/147), private (15.6%, 23/147), and not-for-profit (14.3%, 21/147) sources.

**Table 1:** Summary of study characteristics: *n* = 147

Category	*n* (%)
Study design	
RCT	52 (35.4)
Non-randomized trial	19 (12.9)
Cohort study	38 (25.9)
Case controlled	5 (3.4)
Cross sectional	3 (2.0)
Other (e.g., survey, mixed methods, field study)	30 (20.4)
Study focus	
Hip	40 (27.2)
Knee	78 (53.1)
Both	29 (19.7)
Intervention type	
Other (e.g., patient education, lifestyle intervention, scoring tools)	49 (33.3)
Physiotherapy	29 (19.7)
Surgical	22 (15.0)
Diagnostic	22 (15.0)
Pharmacologic	13 (8.8)
Equipment	12 (8.2)
Intervention category	
Preoperative	27 (18.4)
Intraoperative	31 (21.1)
Postoperative	77 (52.4)
Long term	0 (0.0)
Combined	10 (6.8)
Not applicable	2 (1.4)
Total study population	
Q1: *n* = 2–21	33 (82.5)
Q2: *n* = 22–35	31 (91.2)
Q3: *n* = 36–61	34 (91.9)
Q4: *n* = 62 +	22 (88.9)
Source of funding	
Federal	39 (26.5)
Private sector	23 (15.6)
Not-for-profit	21 (14.3)
None	52 (35.4)
Not specified	12 (8.2)

### Prevalence of spin

Spin was highly prevalent in this study population, with 88.4% (130/147, 95% CI 83.3–93.6) of manuscripts exhibiting at least one of the three predefined spin types. The most common approach involved emphasizing an intervention’s effectiveness or potential benefits rather than its feasibility (81.6%, 120/147, 95% CI 75.3–87.9). In contrast, fewer studies presented statistically non-significant or non-feasible results as effective (27.2%, 40/147, 95% CI 20.0–34.4). However, a majority prioritized statistical significance *over* feasibility (60.5%, 89/147, 95% CI 52.6–68.5). Full details are presented in Table 2.

**Table 2:** Overview of reporting based on the SPIN criteria (*n* = 147)

Overview of SPIN prevalence	Number of studies (n) demonstrating spin (%)	95% CI
Overall: Demonstrated at least 1 type of spin practice	130 (88.4)	83.3, 93.6
Practice 1: A focus on statistical significance rather than feasibility (e.g., secondary outcomes)	89 (60.5)	52.6, 68.5
Practice 2: Presenting non-feasible results (statistically nonsignificant) as feasible or effective	40 (27.2)	20.0,34.4
Practice 3: Emphasizing effectiveness or potential intervention benefits rather than feasibility	120 (81.6)	75.3, 87.9

### Locations of spin reporting

Abstracts and discussions displayed a higher presence of spin, with both frequently incorporating practice 3—framing interventions in terms of potential effectiveness or benefit rather than feasibility. This approach appeared in 68.7% of discussions (101/147, 95% CI 61.2, 76.2) and 56.5% (83/147, 95% CI 48.5, 64.5) of abstracts, whereas other spin types were present in fewer than half of sections. Conclusions showed a marked reduction in overall spin prevalence, declining from 79.6% (117/147, 95% CI 73.1, 86.1) in discussions to 56.5% (83/147, 95% CI 48.5, 64.5), though practice 3 remained the most frequently used strategy. By comparison, spin type 2 (presenting non-feasible results as effective) was consistently the least common across all study components (Table 3).

**Table 3:** Distribution of SPIN by location within a paper

Overview of SPIN instances	*n* (%) with spin type in abstract	95% CI	*n* (%) with spin type in discussion	95% CI	*n* (%) with spin type in conclusion	95% CI
Overall: Any spin practice	116 (78.9)	72.3, 85.5	117 (79.6)	73.1, 86.1	83 (56.5)	48.5, 64.5
Practice 1: A focus on statistical significance rather than feasibility	67 (45.6)	37.5, 53.6	69 (46.9)	38.9, 55.0	22 (15.0)	9.2, 20.7
Practice 2: Presenting non-feasible results as feasible or effective	13 (8.8)	4.3, 13.4	33 (22.5)	15.7, 29.2	12 (8.2)	3.7, 12.6
Practice 3: Emphasizing effectiveness or potential intervention benefits rather than feasibility	83 (56.5)	48.5, 64.5	101 (68.7)	61.2, 76.2	70 (47.6)	39.6, 55.7

### Patterns of spin reporting

**Fig. 2:**
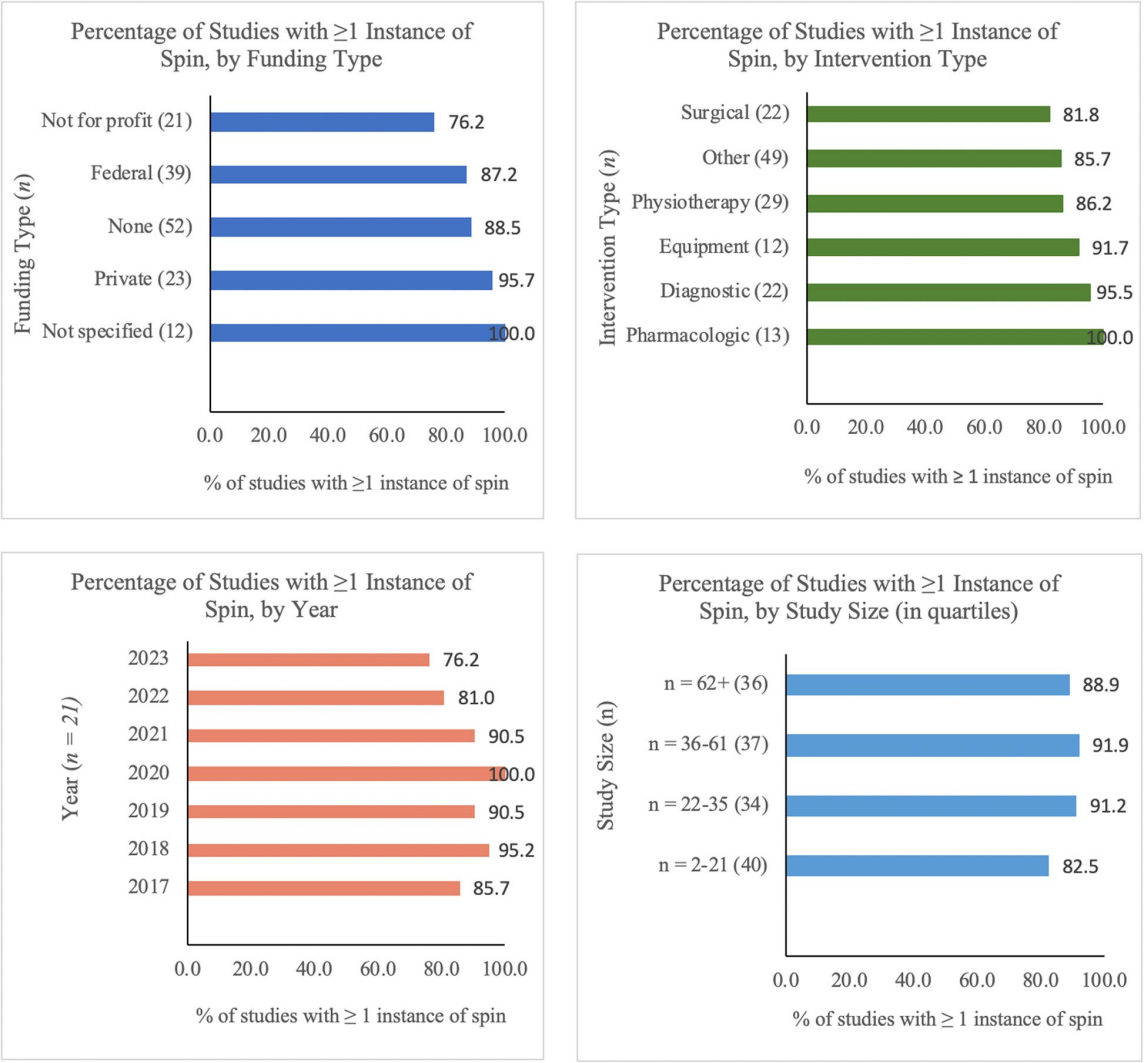
Percentage of pilot and feasibility trials reporting ≥ 1 instance of spin, by funding, intervention, year, and study size

Figure 2 illustrates spin reporting patterns across several study characteristics, including funding source, intervention type, publication year, and sample size. Among studies that disclosed funding information, those backed by a private source exhibited the highest rate of spin at 95.7% (22/23, 95% CI 92.4, 98.9). This was followed closely by federally and non-funded manuscripts (87.2% and 88.5%, respectively). By contrast, works funded by not-for-profit organizations showed the lowest occurrence, at 76.2% (16/21, 95% CI 69.3, 83.1).

All pharmacologic studies (13/13) demonstrated spin, as did 95.5% (21/22, 95% CI 92.1, 98.8) of those involving diagnostic equipment. In comparison, surgical intervention studies showed a lower rate of spin at 81.8% (18/22, 95% CI 75.6, 88.1). No consistent temporal trend was identified, although spin peaked in 2020 (100.0%, 21/21) before declining to 76.2% in 2023 (16/21, 95% CI 69.3, 83.1). Variation was also observed across study sizes: spin appeared less frequently in both small (Q1 *n* = 2–21, 82.5%, 33/40) and large (Q4 *n* ≥ 62, 88.9%, 32/36) samples, while mid-sized studies (Q2 *n* = 22–35 and Q3 36–61) exceeded 90% prevalence.

## Discussion: spin reporting and implications for practice

### Location of spin and overall prevalence

This methodological analysis found a high frequency of spin in pilot and feasibility research on hip and knee arthroplasty, with 88.4% of included manuscripts demonstrating at least one instance. This rate is consistent with prior findings in oncology (75%, 39/52), pharmacovigilance (63%, 63/100), and non-randomized abstracts (84%, 107/128) [7, 13, 16]. However, the use of spin varies considerably across fields. For example, with research in traumatic brain injury and RCT infographics reported much lower prevalence, at 22% (33/150) and 33% (39/119), respectively [9, 14]. A methodological review by Chiu et al. echoed this variability, noting prevalence as low as 9.7% in lung cancer studies and as high as 100% in trials evaluating cardioverter defibrillators [4].

Differences also emerged within sections of individual reports. In this sample, abstracts and discussions sections contained the highest levels of spin (78.9% 116/147, and 79.6% 117/147 of studies) while conclusions showed a marked decline (56.5, 83/147). Similar patterns have been observed in other disciplines; for instance, Lazarus et al. reported spin in 84% of abstracts and Ito et al. found it in 73.1% of main texts from oncology trials. Meanwhile, Boutron et al. observed lower frequencies in parallel-group RCTs, with spin present in just 37.5% of abstracts, 29.2% of main texts and 23.6% of conclusions [5, 12, 14].

Owing to the wide range of reporting styles, spin categorizations, and study populations/sizes examined, establishing a consistent estimate of spin prevalence remains a challenge [4]. The current investigation focused on pilot and feasibility studies, many of which involved smaller sample sizes (*n* < 100) and were intended to inform the development of future definitive studies. Unfortunately, concerns arise when the “pilot” designation is applied post hoc, often to justify small sample sizes or address methodological shortcomings, as documented in the dental literature [17]. When studies adopt this label due to underpowered sample sizes or novel project topics, spin can enhance the perceived significance and readership of smaller projects, possibly contributing to the higher prevalence noted in this study. In this study, spin was most prevalent in studies with sample sizes ranging from 22 to 35 participants, as well as 36–61. However, it should be noted that this project also did not identify a particularly higher presence of spin in studies with the smallest sample sizes in the investigated population (*n* = 2–21). This may reflect fewer opportunities for data over-interpretation, with authors more likely to acknowledge the exploratory nature of smaller-scale projects. At this magnitude, authors may also find themselves faced with stricter guidelines and requirements for publication, promoting a more cautious approach. As a whole, misleading communication can have detrimental effects, leading to misinterpretation of trial conclusions and the formation of inadequate clinical recommendations [18]. When combined with an inadequately designed pilot, spin further undermines the trial’s purpose as a tool for refining and optimizing future project development.

### Type of spin used

Among manuscripts that employed spin, type 3 (emphasizing potential effectiveness or intervention benefits rather than feasibility) was by far the most common, appearing in 81.6% of studies (120/147). This approach reflects a broader trend of overstating favorable outcomes, which has been shown to influence patient perceptions by increasing the likelihood of a treatment option being viewed as beneficial [19]. A systematic review of analgesic RCTs similarly found that suggesting treatment benefit despite non-significant results was among the most prevalent forms of spin (29% of included trials) [20]. Comparable tactics have been observed in other specialties such as wound care, bariatric surgery, and urology, where authors often obscure non-significant findings through vague phrasing and semantic manipulation [21–23]. Rather than reporting feasibility challenges or clearly presenting neutral outcomes, these strategies redirect attention toward speculative trends and implications, ultimately distorting how an intervention is perceived.

This framing may be particularly appealing in pilot and feasibility studies, where demonstrating some form of perceived benefit can help frame a study as “promising” and support its advancement to a full-scale trial. Unlike full-scale RCTs, these early-phase investigations are often seen as “prerequisites” for securing future funding or scaling up. As a result, researchers may feel compelled to highlight findings that appear “useful,” even when feasibility metrics were the intended focus [24]. In support of this, participants in a qualitative study by von Klinggraeff et al. noted that pilot studies labelled as “successful” were more likely to proceed to subsequent trials, while less favorable ones were typically abandoned [24]. These perceived pressures may incentivize the use of spin as a tool to promote study advancement and justify further investment.

### Patterns of spin reporting

Patterns in spin reporting and its potential predictors have been inconsistent. Although 95.7% (22/23) of private/industry-funded studies in this review exhibited at least 1 instance of spin compared with 76.2% (16/21) of their not-for-profit counterparts, no definite associations have been established in the literature. For example, a meta-analysis of 1110 spin-focused studies by Chiu et al. found no increased likelihood of spin in industry-sponsored research relative to non-industry-funded work (RR 1.08, 95% CI 0.87, 1.34) [4].

Even so, evidence from specific fields has suggested otherwise. In neurosurgery, a review by Khan et al. reported that industry-sponsored trials were 23 times more likely to present favorable outcomes, potentially due to factors such as publication agreements or preferential funding [25]. Likewise, in wound care, 89% of industry-funded RCTs abstracts demonstrated spin compared to 60% of those with non-profit support [23]. While the role of funding remains difficult to investigate in the context of heterogeneous study designs, researchers should remain mindful of the potential bias it may introduce to intent and framing of a study.

Spin was also most prevalent in trials investigating pharmacologic interventions, appearing in 100% (12/12) of such studies. Although evidence on the influence of intervention type remains mixed, several plausible explanations have been proposed regarding its potential to foster higher spin use. Drug trials are often commercially sponsored, especially when conducted by or in manufacturing companies, introducing potential conflicts of interest [26]. At the same time, these studies frequently involve complex designs with multiple endpoints and secondary outcomes, creating more opportunities for selective emphasis. External pressure from stakeholders, including regulatory agencies and review committees, may also encourage the presentation of “positive” or actionable results. Even so, trials focused on surgical interventions demonstrated the lowest prevalence of spin (81.8%) despite being subject to many similar pressures that may be faced by their pharmacological counterparts. A possible emphasis on objectives focused on technical process feasibility and safety compared to efficacy or non-inferiority may contribute to this observation, as well as different target endpoints. No consistent patterns in spin frequency were observed when grouped by publication year or sample size.

### Implications for practice

The presence of spin in pilot and feasibility literature on hip and knee arthroplasty introduces opportunities for bias and misrepresentation, ultimately distorting the field’s understanding of emerging interventions. Advancement requires both favorable and unfavorable results to shape future trial design and minimize research redundancy. Yet, as seen in cervical arthroplasty trials registered on clinicaltrials.gov, there is a tendency to highlight outcomes with perceived clinical impact, while less impressive results often go unpublished [27].

This dynamic is particularly concerning in arthroplasty, where 87.5% of industry-funded studies report favorable conclusions, and 75% of hip and knee implant trials receive commercial sponsorship. These conditions create an environment highly susceptible to potential influences promoting spin, increasing the risk that results are selectively emphasized to portray interventions in a more favorable light [28, 29]. As noted earlier, such framing can shape reader perceptions, increasing the likelihood that treatments are rated as beneficial and driving greater interest in full-text review [19, 30]. The cumulative effect is a decline in the true clinical applicability of findings, which may ultimately hinder informed decision making by clinicians, caregivers, and patients.

## Discussion: recommendations, strengths, and limitations

### Recommendations to mitigate spin

To mitigate the risks associated with spin, clear reporting frameworks are essential. This is where clear reporting guidelines for pilot and feasibility trials, such as the CONSORT extension, can help to guide outcomes reporting, directing attention to areas such as study powering that may be easily overlooked. Prospective trial registration (e.g., through ClinicalTrials.gov) that explicitly outlines feasibility objectives pertaining to feasibility metrics can also hold authors accountable to predetermined aims, rather than switching the emphasis to more appealing post hoc narratives. Finally, authors should explicitly study limitations and communicate them transparently. This action serves as a reminder to interpret findings with caution and an understanding of limits.

### Strengths and limitations

Several limitations should be acknowledged from this study. Spin can be interpreted and classified in various ways, and this project specifically adapted the checklist from Boutron et al. and McKechnie et al. to suit the context of pilot/feasibility trials [5, 11]. However, the heterogeneity in classification tools across the spin-focused literature, some of which included more than three spin categories, may limit direct comparisons and contribute to differing estimates of prevalence. Projects using broader definitions may naturally report higher rates of spin than those using a narrower framework, as was done here. In addition, while three independent reviewers conducted a data extraction process with strong interrater agreement, the process still involved an element of subjectivity, particularly in evaluating the more nuanced cases of spin. Furthermore, because this was a descriptive analysis, potential influencing factors—such as funding source, intervention type, publication year, and sample size—were evaluated solely in terms of frequency. Additional statistical analysis, including logistic regression may be beneficial in understanding predictors of spin. While PubMed was the only database used in this methodological analysis, larger-scale systematic reviews and meta-analyses may also use databases such as Embase to obtain a larger sample size for review. Within the context of the search strategy, the timeframe of 2017–2023 may not fully encompass more recent studies, that may influence the observed estimates and patterns of spin reporting. Looking ahead, efforts to develop a more unified framework for classifying and recognizing spin may benefit both researchers and readers. Standardized approaches would also enable more reliable cross-study comparisons and help clarify patterns across different research domains and trial designs.

## Conclusions

Consistent with trends across other healthcare fields, pilot/feasibility trials in hip and knee arthroplasty employ high levels of spin reporting, particularly in abstracts and main texts. Spin was more prevalent in industry-funded and pharmacologic projects, although the specific influence of study type and funding source remains uncertain. Given the role of these trials in shaping subsequent research and clinical innovation, the presence of spin may distort perceptions of intervention utility and feasibility. Improved adherence to reporting guidelines such as the CONSORT extension for pilot trials, along with clear communication of study limitations and feasibility objectives, will be critical to enhancing transparency and minimizing interpretive bias in this literature.

## Data Availability

All datasets used and/or analyzed during the current study are available from the corresponding author on reasonable request.

## Appendix 1

### PubMed search strategy

**Table:**

	Search terms	Results
#1	("Feasibility studies"[MeSH Terms] OR "feasibility project*"[Text Word] OR "feasibility trial*"[Text Word] OR "feasibility trials"[Text Word] OR "feasibility stud*"[Text Word]) AND (2017:2023[pdat])	36 414
#2	("Pilot projects"[MeSH Terms] OR "pilot project*"[Text Word] OR "Pilot projects"[Text Word] OR "pilot trial*"[Text Word] OR "pilot trials"[Text Word] OR "pilot stud*"[Text Word]) AND (2017:2023[pdat])	70 673
#3	("arthroplasty, replacement, knee"[MeSH Terms] OR "knee replacement*"[Text Word] OR "knee replacements"[Text Word] OR "knee arthroplast*"[Text Word]) AND (2017:2023[pdat])	22 216
#4	("arthroplasty, replacement, hip"[MeSH Terms] OR "hip replacement*"[Text Word] OR "hip replacements"[Text Word] OR "hip arthroplast*"[Text Word]) AND (2017:2023[pdat])	20 347
#5	#1 OR #2	102 447
#6	#3 OR #4	37 735
#7	#5 OR #6	426
